# Epidemic features affecting the performance of outbreak detection algorithms

**DOI:** 10.1186/1471-2458-12-418

**Published:** 2012-06-08

**Authors:** Jie Kuang, Wei Zhong Yang, Ding Lun Zhou, Zhong Jie Li, Ya Jia Lan

**Affiliations:** 1 Department of Occupational Health, West China School of Public Health, Sichuan University, 17 South Section 3 Renmin Road, Chengdu, Sichuan 610041, China; 2 Key Laboratory of Surveillance and Early-warning on Infectious Disease, Chinese Center for Disease Control and Prevention (China CDC), 155 Changbai Road Changping District, Beijing, 102206, China

**Keywords:** Epidemic feature, Outbreak detection algorithms, Performance, Automated infectious disease surveillance

## Abstract

**Background:**

Outbreak detection algorithms play an important role in effective automated surveillance. Although many algorithms have been designed to improve the performance of outbreak detection, few published studies have examined how epidemic features of infectious disease impact on the detection performance of algorithms. This study compared the performance of three outbreak detection algorithms stratified by epidemic features of infectious disease and examined the relationship between epidemic features and performance of outbreak detection algorithms.

**Methods:**

Exponentially weighted moving average (EWMA), cumulative sum (CUSUM) and moving percentile method (MPM) algorithms were applied. We inserted simulated outbreaks into notifiable infectious disease data in China Infectious Disease Automated-alert and Response System (CIDARS), and compared the performance of the three algorithms with optimized parameters at a fixed false alarm rate of 5% classified by epidemic features of infectious disease. Multiple linear regression was adopted to analyse the relationship of the algorithms’ sensitivity and timeliness with the epidemic features of infectious diseases.

**Results:**

The MPM had better detection performance than EWMA and CUSUM through all simulated outbreaks, with or without stratification by epidemic features (incubation period, baseline counts and outbreak magnitude). The epidemic features were associated with both sensitivity and timeliness. Compared with long incubation, short incubation had lower probability (β* = −0.13, P < 0.001) but needed shorter time to detect outbreaks (β* = −0.57, P < 0.001). Lower baseline counts were associated with higher probability (β* = −0.20, P < 0.001) and longer time (β* = 0.14, P < 0.001). The larger outbreak magnitude was correlated with higher probability (β* = 0.55, P < 0.001) and shorter time (β* = −0.23, P < 0.001).

**Conclusions:**

The results of this study suggest that the MPM is a prior algorithm for outbreak detection and differences of epidemic features in detection performance should be considered in automatic surveillance practice.

## Background

Infectious diseases remain the major causes of morbidity and mortality in China despite substantial progress in their control [1]. The outbreaks of infectious diseases pose serious threats on public health. Early detection of aberration and rapid control actions, which the Chinese Ministry of Health has taken as important strategies for emergency infectious disease prevention and control [2], are prerequisites for preventing the spread of outbreaks and reducing the morbidity and death caused by diseases. Therefore, China Infectious Disease Automated-alert and Response System (CIDARS) was successfully implemented and began to operate nationwide in 2008 [3].

At the end of 2010, analysis results of the operation of CIDARS in nationwide showed that a large number of outbreaks of infectious diseases could be timely detected, but it was also found that there were many of false-positive signals; large differences existed between outbreak signal counts and final identified outbreaks in different diseases; the detection performance was poor in those diseases which had more case reports and fewer outbreaks [4]. These issues prompted us that epidemic features of infectious disease may affect outbreak detection performance.

Several studies have described the determinants of outbreak detection performance, including: system factors (representativeness, outbreak detection algorithms and algorithm-specifics), outbreak characteristics (outbreak size, shape of the outbreak signal and time of the outbreak) [5-8]. Understanding the differences these determinants make in detection performance can help public health practitioners improve the automated surveillance system, thus raising detection capabilities. Recently, extensive researches have explored novel algorithms to improve the performance of outbreak detection [9-13], but evidence on how epidemic features impact on detection performance is still limited.

To address this limitation, our study aimed to explore the influence of epidemic features (incubation period, baseline counts and outbreak magnitude) on algorithms’ detection performance. Findings of this study may help public health surveillance practitioners understand the detection performance of algorithms under these epidemic features and improve the implementation of automated surveillance.

## Methods

**Figure 1:**
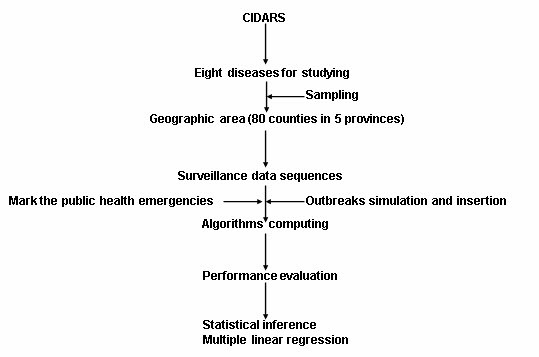
**The flowchart of data processing**.

Figure 1 presents the flow of data processing in this study. First, we selected eight notifiable infectious diseases in CIDARS for studying. Second, we sampled ten counties for each infectious disease. Third, we exacted the surveillance data sequences in 2005–2009 from CIDARS. Fourth, we marked the public health emergencies and injected simulated outbreaks in data sequences. Fifth, we ran the three outbreak detection algorithms. Sixth, we computed the sensitivity and timeliness of the three outbreak detection algorithms. Finally, statistical inference and multiple linear regression were used to compare the performance and examine the relationship of sensitivity and timeliness with epidemic features.

### Data sources

The infectious disease data were extracted from CIDARS. CIDARS was developed basing on the existing data from National Disease Surveillance Reporting and Management System on 28 diseases that are outbreak-prone and require prompt action. The 28 diseases were classified into two types according to severity, incidence rate and importance [3]. Type 1 diseases includes nine infectious diseases characterized with higher severity but lower incidence and are analysed using fixed-threshold detection method. For type 2 diseases (19 more common infectious diseases) , we selected eight diseases (dysentery, scarlet fever, mumps, measles, malaria, typhoid, encephalitis B and hepatitis A) which represented three routes of transmission(respiratory, oral-fecal and vector-borne). Five provinces were sampled for eight diseases(dysentery in Hunan, scarlet fever, measles in Xinjiang, mumps in Chongqing, malaria, typhoid, encephalitis B in Yunnan, hepatitis A in Guizhou), where the respective disease had high incidence and became important local public health problems. Then we randomly sampled 10 counties from the selected provinces for each disease, and obtained their actual daily number of reported cases in 2005–2009. Data from 2005 to 2007 were used as baseline, while data from 2008 to 2009 were used to evaluate the algorithms.

Strategy of inserting simulated outbreaks was used to evaluate the detection performance. To prevent public health emergency confounding the evaluation, we got the records of public health emergency in the corresponding counties from Emergency Public Reporting System [14].

### Outbreak detection algorithms

To date many outbreak detection algorithms can be used for temporal data [15-19]. Considering that we collected five years data, using the same periods’ historical data as baseline is appropriate, to some extent, can reduce the seasonal and day-of-week variation in the baseline. However, for using this, the regression and ARIMA models may subject to certain restrictions, as the steps in their processing require recent continuous time interval to calculate expected statistic. So we chose two most commonly used statistical process control algorithms (EWMA, CUSUM) and a non-parameter algorithm(MPM) which enable the application of the same periods’ historical data in theory.

For EWMA, the current time-series value is replaced by a weighted average of the recent values. If the observed values were assumed for Poisson distribution,The smoothed daily count was calculated as

(1) Zt= λ X¯t+·1− λ ·Zt-1

and the upper control limit(UCL) was calculated as

(2) UCL=μ+·kσ λ 2− λ

In the algorithm, the λ (0 <  λ  < 1) was the weighting factor, k was the control limit coefficient [20].

Cumulative Sum (CUSUM) has been used to detect outbreaks of infectious diseases in recent decades [21]. Two parameters k and h are involved. k refers to the pre-specified reference value and h the decision boundary. Here, μ_t_ is the sample mean and σ_t_ the standard deviation of the reference values. When we denote x_t_ the count per day, then the CUSUM statistic is defined as

(3) $$S_{t}=\text{max}\left(0,{\text{ S}}_{t}{}_{-1}+\left(x_{t}-\left(\mu_{t}+\text{ k}\sigma_{t}\right)/\sigma_{t}\right)\right)$$

If the CUSUM statistic is larger than h, then the current day is considered as a possible outbreak.

The MPM uses previous several years (such as 3–5 years) over the same period as baseline data, setting a percentile of baseline data as a detection parameter c. If the current day counts x_t_ is greater than the detection parameter's corresponding percentile (P_c_), outbreak signal is produced [22].

### Algorithm parameters

To obtain the optimized parameter values of the three algorithms, we used R software [23] generating two-year Poisson distribution sequences with five daily average counts levels (0.1, 0.5, 1.0, 2.0, 5.0). We set a fixed false alarm rate of 0.05 (an average of one false alarm every twenty days) by applying each algorithm to these five sequences without any added outbreak signals, and determining the parameters that would yield an average of one false alert every twenty days (see Additional file 1: Table S1).

### Baseline data

**Figure 2:**
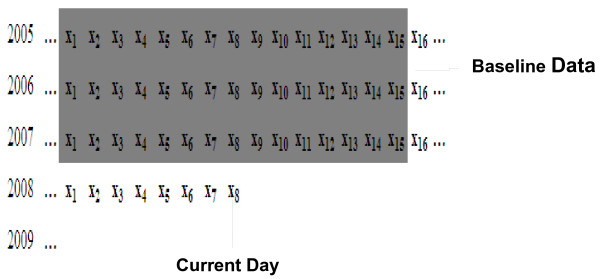
Example of baseline data for EWMA, CUSUM, and MPM.

**Figure 3:**
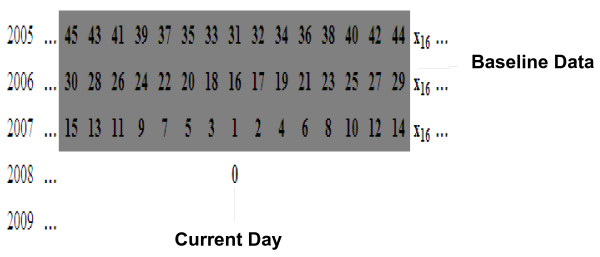
**Example of the order number of baseline data for EWMA**.

We used baseline data from 2005 to 2007, in which we use the corresponding day, the seven previous days and the seven later days. These summed up to a total of 45 days (Figure 2). The EWMA algorithm was associated with the order day of baseline data (Figure 3).

### Outbreak simulation and insertion

**Figure 4:**
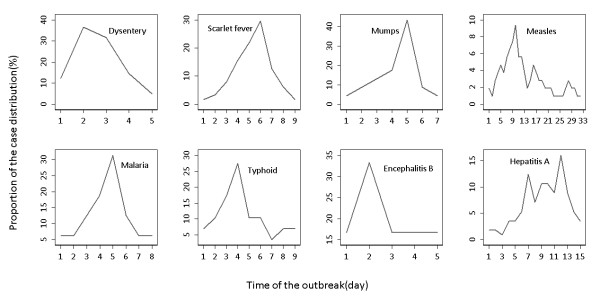
**The proportion of daily case distribution for eight outbreaks**.

We generated outbreak signals by simulation. We selected real outbreak events reported in the literatures for each disease [24-31], and calculated the proportion of daily case distribution (Figure 4). Next, we simulated four outbreak magnitudes (0.5, 1.0, 2.0, 3.0), which meant that the increase cases of outbreak signals are the corresponding multiple of the baseline number of cases.

As the baselines were required to use the same period of history (seven days before and after the current day), to increase the stability of the calculation, we made 10th-day the insertion date of outbreak signals every month. If the intervals had public health emergency, then skipped the insertion. As the outbreak duration of measles was more than one month, the outbreak signals were injected every two months. Finally, we had eight kinds of outbreak signals, each disease included ten counties, and each county had four outbreak magnitude test datasets, which summed up to 320 test datasets. In theory, 7,200 outbreak signals could be inserted. Excluding the public health emergencies, the actual outbreak signals inserted were 7,088 (Table 1).

**Table 1:** Summary the number of injected outbreak signals

**Diseases**	**County**	**Outbreaks should be injected**	**Public health emergencies**	**Actual injected outbreaks**
**Dysentery**	10	960	1	956
**Scarlet fever**	10	960	3	948
**Mumps**	10	960	20	880
**Measles**	10	480	0	480
**Malaria**	10	960	0	960
**Typhoid**	10	960	1	956
**Encephalitis B**	10	960	3	948
**Hepatitis A**	10	960	0	960
**Total**	80	7200	28	7008

### Incubation classification

According to the minimum, maximum, average incubation period of eight diseases [32], eight diseases were clustered into three categories by K-means clustering method: the short incubation period disease (dysentery, scarlet fever), the medium incubation period disease (mumps, measles, malaria, typhoid, encephalitis B) and the long incubation period disease (hepatitis A) (Table 2).

**Table 2:** Classification the incubation of diseases with K-means clustering

**Diseases**	**Incubation period(day)**			**Clustering classification**
	**Minimum**	**Maximum**	**Average**	
**Dysentery**	1	7	2	Short
**Scarlet fever**	2	12	4	Short
**Mumps**	8	30	18	Medium
**Measles**	6	21	10	Medium
**Malaria**	7	30	17	Medium
**Typhoid**	5	21	9	Medium
**Encephalitis B**	10	15	12	Medium
**Hepatitis A**	15	45	30	Long

### Performance evaluation

Performance comparisons were based on three indicators: sensitivity (the proportion of outbreaks the algorithm detected), timeliness (the difference between the date of the first true alarm and the beginning date of the outbreak) and false alarm rate (the proportion of non-outbreak days on which the algorithm signal an alarm).

For more informative comparisons of the performance in different determinants, we plotted sensitivity-timeliness curve [33], which measured the proportion of outbreaks that an algorithm detected within several days from the start of the outbreak.

### Statistical inference and multiple linear regression

For each of the eight diseases in each of the ten counties on each of the four out- break magnitudes, we computed the sensitivity, timeliness and false alarm rate across all 320 analysis runs. We used ANOVA to test for significant difference in the algorithms’ sensitivities and timeliness. The Bonferroni correction was applied for multiple comparisons to control the family wise error rate. The significance level α was 0.05. Finally, multiple linear regression was run to understand the relationship of the algorithms sensitivities and timeliness with disease incubation, baseline counts and outbreak magnitude. All the analyses were performed using R software.

## Results

### Algorithm performance

The respective proportion of detected outbreak signals of EWMA, CUSUM, MPM were 56.02%, 58.72%, 69.71%. Overall, the MPM was more sensitive than EWMA, CUSUM. In timeliness, the average lag days of all outbreak signals were: EWMA 2.40, CUSUM 2.52 and MPM 1.65. Compared with EWMA and CUSUM, the MPM needed shorter time to detect outbreak signals (P <0.001). The false alarm rate of EWMA, CUSUM and MPM were 5.09, 5.85 and 4.93. The false alarm rate did not differ in three algorithms (P = 0.153) (Table 3).

**Table 3:** Summary the performance of outbreak detection algorithms

**Performance indicators**	**Mean**	**95% confidence interval**	**F value**	***P*-value**
**Sensitivity(%)**				
EWMA	56.02	53.08-58.95	22.04	<0.001
CUSUM	58.72	55.81-61.63		
MPM*	69.71	66.46-72.96		
**Timeliness(Day)**				
EWMA	2.40	2.20-2.62	20.28	<0.001
CUSUM	2.52	2.31-2.73		
MPM*	1.65	1.45-1.85		
**False alarm rate(%)**				
EWMA	5.09	4.39-5.78	1.88	0.153
CUSUM	5.85	5.08-6.63		
MPM	4.93	4.28-5.58		

* p < 0.001.According to Bonferroni’s procedure, the family wise error rate was 0.05,divided by the number of test. The significance level αfor an individual test was 0.05/3,being 0.017.

### Sensitivity-timeliness plot

**Figure 5:**
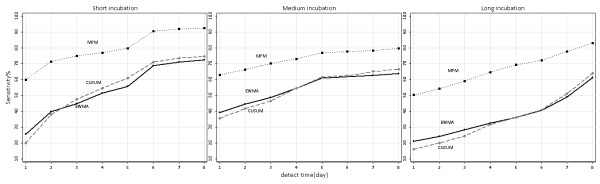
**Sensitivity versus timeliness for three detection algorithms in three incubation categories.** EWMA, exponentially weighted moving average; CUSUM, cumulative sum; MPM, moving percentile method.

**Figure 6:**
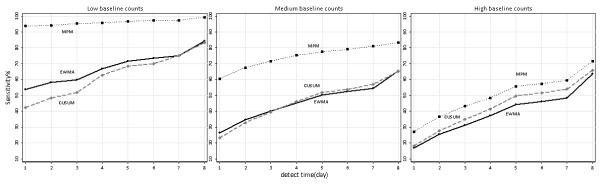
**Sensitivity versus timeliness for three detection algorithms in three baseline counts levels.** EWMA, exponentially weighted moving average; CUSUM, cumulative sum; MPM, moving percentile method.

Figures4, 5, 6 were sensitivity-timeliness plot to compare three algorithms performance in different incubation categories, baseline counts levels and outbreak magnitudes.

In the three incubation categories, the MPM had a higher probability of detecting the outbreak than CUSUM and EWMA within all days from the start of the outbreak; the sensitivity changes of CUSUM and EWMA over time were very close. In short incubation, the sensitivity of MPM reached 90% at the sixth day of a outbreaks start. The long incubation disease had a poorer sensitivity than short and medium incubation disease (Figure 5).

We used the 33.33, 66.67 percentiles with the cuts off 0.05, 0.2 to divide average baseline counts into low, medium and high levels. At the three levels, the MPM had a higher probability of detecting the outbreak than CUSUM and EWMA within all days from the start of the outbreak; the sensitivity of CUSUM and EWMA was very closely changed over time. The sensitivity of low baseline counts was better than medium and high levels, with the MPM reaching higher than 90% (Figure 6).

**Figure 7:**
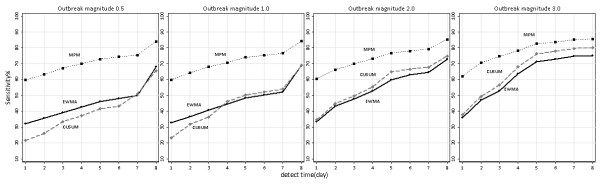
**Sensitivity versus timeliness for three detection algorithms in four outbreak magnitudes.** EWMA, exponentially weighted moving average; CUSUM, cumulative sum; MPM, moving percentile method.

In the four outbreak magnitudes, the MPM had a higher probability of detecting outbreak than CUSUM and EWMA within all days from the start of the outbreak; the CUSUM method had a better sensitivity than EWMA in 2.0,3.0 magnitude (Figure 7).

### Multiple linear regression

The variable coding in multiple linear regression (Table 4).

**Table 4:** Variable coding in multiple linear regression

**Variable**	**Variable coding**
**Dependent Variable**	
Sensitivity or Timeliness	Actual value
**Independent Variable**	
Algorithm	1 CUSUM
	2 MPM
	3 EWMA
Incubation	1 Short
	2 Medium
	3 Long
Baseline counts	Actual value
Outbreak magnitude	0.5
	1.0
	2.0
	3.0

The results of multiple linear regression showed that algorithms, incubation period, baseline counts and outbreak magnitude were associated with both sensitivity (Table 5) and timeliness (Table 6).

**Table 5:** Summary of Multiple Regression Analysis for Variables Predicting Sensitivity

**Variable**	***β***	***SE***	β*	***P*-value**
**Algorithms**				
CUSUM	2.70	1.74	0.04	0.121
MPM	13.69	1.74	0.21	<0.001
EWMA(reference )				
**Incubation**				
Short	−9.68	2.47	−0.13	<0.001
Medium	−6.97	2.21	−0.11	0.002
Long(reference )				
**Baseline counts**	−12.01	1.38	−0.20	<0.001
**Outbreak magnitude**	16.11	0.66	0.55	<0.001
***R***^***2***^ **= 0.39**				
***F*=125.85**				<0.001

β* standard regression coefficient.

**Table 6:** Summary of Multiple Regression Analysis for Variables Predicting Timeliness

**Variable**	***β***	***SE***	β*	***P*-value**
**Algorithms**				
CUSUM	0.10	0.13	0.02	0.420
MPM	−0.76	0.13	−0.17	<0.001
EWMA(reference )				
**Incubation**				
Short	−2.80	0.18	−0.57	<0.001
Median	−2.42	0.16	−0.56	<0.001
Long(reference )				
**Baseline counts**	0.61	0.11	0.14	<0.001
**Outbreak magnitude**	−0.46	0.05	−0.23	<0.001
***R***^***2***^ **= 0.28**				
***F*=73.29**				<0.001

β* standard regression coefficient.

As it was illustrated in Table 5, the algorithms, incubation period, baseline counts and outbreak magnitude had a statistically significant relationship with sensitivity. MPM had a higher probability of detecting outbreaks compared with EWMA (β* = 0.21, P < 0.001). Short(β* = −0.13, P < 0.001) and Medium(β* = −0.11, P = 0.002) incubation had a lower probability of detecting the outbreaks compared with long incubation. The lower the baseline counts, the higher probability (β* = −0.20, P < 0.001). The larger outbreak magnitude, the higher probability (β* = 0.55, P < 0.001).

The algorithms, incubation period, baseline counts and outbreak magnitude had a statistically significant relationship with timeliness. MPM needed shorter time to detect the outbreaks compared with EWMA(β* = −0.17, *P* < 0.001). Short(β* = −0.57, *P* < 0.001) and Medium(β* = −0.56, *P* < 0.001) incubation needed shorter time to detect the outbreaks compared with long incubation. The higher baseline counts, the longer time to detect the outbreaks (β* = 0.14, *P* < 0.001). The larger outbreak magnitude, the shorter time (β* = −0.23, *P* < 0.001) (Table 6).

## Discussion

Many determinants affect the performance of outbreak detection in automated surveillance, and knowing about how these factors influence the detection performance can help to improve automated surveillance system. In this study, we compared the performance of three outbreak detection methods by adding simulated outbreaks to actual daily counts of notifiable infectious diseases in CIDARS and examined the relationship of the detection performance with disease incubation, baseline counts and outbreak magnitude.

In algorithms’ detection performance, we found MPM had better performance than the EWMA, CUSUM methods. In theory, MPM method is simple, with fewer parameters and without the limit of the overall distribution of monitoring data. The results showed that the performance of MPM was stable under different test conditions, which indicated that the MPM method has a broad scope of application. These advantages prompted that this method should be first considered when designing an automatic disease surveillance system.

Consistent with previous evaluations of outbreak detection algorithms [5,34-36], the multiple linear regression results found that the ability to detect outbreaks was better with lower baseline counts and larger magnitude. While previous studies inserted the actual counts with fixed outbreak case numbers, our study inserted the actual counts with outbreak case numbers based on the proportion of case distribution of simulated outbreaks.

Our study examined how different incubation periods affect the detection performance. There are three indicators(minimum, maximum and average of incubation period) to describe incubation, and these three indicators are closely related. To date there is still no definitely way of classifying the incubation period with these three indicators. So we tried the K-means clustering method to classify eight types of disease into three categories. The regression results showed that diseases of long incubation period had a higher sensitivity, but needed more time to detect than those of short and medium incubation period. Generally, the outbreaks of short incubation diseases occur ferociously and transiently, which are more easily to be missed by algorithms. The outbreaks of long incubation diseases, however, occur with longer duration, and can be detected by algorithms more accurately. As the early detection of outbreaks is important, additional work is still needed for timely detection long incubation diseases.

The biggest challenge for the evaluation of outbreak detection algorithms is to obtain a sufficiently large number of outbreak data with which to measure sensitivity and timeliness [33]. Injecting geometric shaped spikes into real surveillance data is a feasible approach [5,36-39]. In this study, in addition to inserting literatures-based simulated outbreak, we also used the public health emergency data. This method provided a solution on the issues where the simulated data may completely detach from real outbreaks and outbreaks in the real surveillance data may interfere with the performance evaluation.

We set a fixed false alarm rate of 0.05, using simulation method to obtain the optimized parameters of different daily average counts data, and focused on the evaluation based on sensitivity and timeliness, which could make the comparison more clear.

There are several limitations to our study that should be taken into consideration. First, eight types of diseases clustering into three categories may not have a good representation. Second, the simulated outbreaks of eight diseases based on literature have some limitations to reflect the complexity of real outbreaks. Third, as only using two-year test datasets, we inserted a limited number of simulated outbreaks, which, to some extent, may affect the stability of the evaluation. In addition, due to the large amounts of computation in this study, we only compared three detection algorithms, so the evaluation of other algorithms needed to be further carried out.

## Conclusions

The results of this study show that the MPM has better detection performance than EWMA, CUSUM. It can be considered as a prior algorithm for automatic infectious disease outbreak detection. Infectious disease outbreak detection performance varies with incubation period, baseline counts and outbreak magnitude. This suggests that the actual automatic infectious disease surveillance practice should take epidemic features into consideration, and select the appropriate algorithm to improve detection performance.

## Competing interests

The authors declare that they have no competing interests.

## Authors’ contributions

JK and YJL designed the study, JK conducted the study and drafted the manuscript, WZY, DLZ and ZJL participated in the design of the study and involved in revising the manuscript. All authors have read and approved the final manuscript.

## Pre-publication history

The pre-publication history for this paper can be accessed here:

http://www.biomedcentral.com/1471-2458/12/418/prepub

## Supplementary Material

Additional file 1 The optimized parameters for three algorithms, at an false alarm rate of 5%. Click here for file
