# Supplementary material for: Epidemic features affecting the performance of outbreak detection algorithms
Source: BMC Public Health. 2012 Jun 8;12:418. doi: 10.1186/1471-2458-12-418 (PMC3489582; doi:10.1186/1471-2458-12-418)
Supplement: Additional file 1 — The optimized parameters for three algorithms, at an false alarm rate of 5%. [file 1471-2458-12-418-S1.pdf]

Additional file 1: The optimized parameters for three algorithms, at a false alarm rate of 5%

Baseline counts	CUSUM		EWMA		MPM	False alarm rate (%)
	h	k	λ	k	c	
0.1	1.20	1.7	0.25	7.40	0.92	4.70～4.98
0.5	0.50	1.7	0.25	6.30	0.95	5.12～5.26
1.0	0.26	1.7	0.25	5.52	0.95	4.70～5.26
2.0	0.26	1.7	0.25	5.28	0.95	4.98～5.39
5.0	0.20	1.5	0.25	4.70	0.95	5.12～5.39
